# Molecular integrity and global gene expression of breast and lung cancer stem cells under long-term storage and recovery

**DOI:** 10.1007/s10561-012-9315-3

**Published:** 2012-05-18

**Authors:** Feridoun Karimi-Busheri, Victoria Zadorozhny, Ewa Carrier, Habib Fakhrai

**Affiliations:** 1 Stem Cell Department, NovaRx Corporation, 6828 Nancy Ridge Drive, San Diego, CA 92121 USA; 2 Present Address: Department of Oncology, University of Alberta, Edmonton, Canada

**Keywords:** Breast cancer stem cells, Lung cancer stem cells, Mammospheres, Lungospheres, Biomarkers, Cryopreservation

## Abstract

Cryopreservation is a common procedure widely used in biological and clinical sciences. Similar protocols are also applied in preserving cancer stem cells, a field with high promises and challenges. Specific cell surface membrane proteins are considered to be biomarkers of cancer stem cells and they may play a critical role in differentiating stem cells from non stem cells. We have looked at the possible effect of long-term cryopreservation on the molecular integrity of breast MCF7 and lung, A549 and H460, cancer stem cells and to assess if these cells are more sensitive to long-term storage process. We analyzed the expression of CD24 and CD38 as two potent biomarkers of lung cancer stem cells and EpCAM and ALDH that are used as biomarkers of a wide range of cancer stem cells. We also selected three genes essential for the normal functioning of the cells, Fos, MUC1, and HLA. Our results indicate a pattern of down-regulation in the expression of the genes following freezing, in particular among cell surface marker proteins. Global gene expression of the post-thaw breast and lung cancer stem cells also reveals a significant down-regulation in freeze-thaw cells independent from each other. Analyzing the canonical pathways between two populations reveals a significant alteration in the gene expression of the pathways involved in cell cycle, mitosis, and ataxia telangiectasia mutated pathways. Overall, our results indicate that current protocols for long-term storage of lung and breast cancer stem cells may substantially influence the activity and function of genes.

## Introduction

Cryopreservation has played a significant role in the advancement of modern science, in particular in biological science, since its application in mid twentieth century by James Lovelock (Mazur 1970). Despite physical and biological stresses to the cells and tissues during long term storage (Bischof et al. 2002) almost every field from basic to clinical sciences and from embryonic stem cells to regenerative medicine has benefitted greatly by cryoprotectants and cryopreservation techniques (Pegg 2002; Manipalviratn et al. 2006; Woods et al. 2004; Berz et al. 2007; Ware et al. 2005).

Cancer stem cells also known as tumor-initiating cells is an emerging field of study that has extensively expanded our vision on cancer development, progression and metastasis (Karimi-Busheri et al. 2010a, b; Pardal et al. 2003; Zhang and Rosen 2006; O’Brien et al. 2009; Monteiro and Fodde 2010). These cells have been isolated from almost all malignancies including hematopoietic, breast, brain, pancreas, and prostate cancer (Notta et al. 2011; Al-Hajj et al. 2003; Eyler et al. 2011; Lonardo et al. 2010; Lang et al. 2009). Cells associated to stem cells-like properties are capable of self-renewal and differentiation, and are resistant to conventional chemo- and radiation therapy (Milas and Hittelman 2009; Rich and Eyler 2008; Kasprzycka et al. 2006). Cancer stem cells have been shown to produce cytokines, chemokines, angiogenic factors (Levina et al. 2008; Hermann et al. 2007), and possess up-regulated signaling cascades essential for cancer metastasis, including hedgehog, epidermal growth factor receptor, NOTCH, and Bmi-1 (Mimeault and Batra 2010; Simeone 2008).

The application of cryopreservation techniques and the impact of freezing and thawing on these cells have been poorly investigated. Only a handful papers have directly addressed this issue (Karimi-Busheri et al. 2010a, b; Chong et al. 2009) and more investigation with proper improvements or modifications being tested and verified on cancer stem cells as a new breed of cells is needed.

Here we report an extensive study on the impact of long term storage of H460 and A549 lung and MCF7 breast cancer stem cells on the molecular integrity of the cells. We present data on the effect of freezing and thawing on biomarkers of cancer stem cells and other pathways. For the first time, to our knowledge, we also provide data on the alterations in the global gene profiling of cancer stem cells under cryopreservation environments and discuss if the changes are biologically meaningful and universal.

## Materials and methods

### Cell line, chemicals, and antibodies

The H460 (HTB-177) and A549 (CCL-185) lung and MCF7 (HTB-22) breast cancer cell lines were purchased from American Type Culture Collection (ATCC, Rockville, MD) and cultured in recommended ATCC media. Modified Eagle Medium/F12 (DMEM) was purchased from SAFC Biosciences (Lenexa, KS), sodium bicarbonate sodium pyruvate were purchased from VWR (West Chester, PA), Penicillin/Streptomycin and B27 Supplement were purchased from Invitrogen (Life Technologies, Carlsbad, CA), and basic fibroblast growth factor (bFGF) was obtained from Millipore Inc. (Billerica, MA). Cancer stem cells were grown on low binding suspension flasks and plates purchased from Sarstedt Inc. (Newton, NC). CD38 phycoerythrin (PE), CD24 Alexa Flour 488, PE anti-human CD326 (EpCAM), and anti-human HLA-A,B,C antibody were obtained from BioLegend (San Diego, CA) and mouse anti-human CD227 (MUC1) FITC from BD biosciences (San Diego, CA). All other chemicals not specifically mentioned were from Sigma-Aldrich (St. Louis, MO).

### Isolation and characterization of lung cancer stem cells (lungospheres)

Lungospheres grow as non-confluent cells of H460 and A549 lung cancer cell lines (Karimi-Busheri et al. 2010a, b). The authentication of both cell lines were validated by the amplification of short tandem repeated DNA sequences using PowerPlex 1.2 System (Promega, Madison WI) according to manufacturer instructions. Analysis of the data on Applied Biosystems ABI Prism 310 Genetic Analyzer confirmed the origin of the cell lines and perfectly matched with the parental cell lines obtained from ATCC (Karimi-Busheri et al. 2012).

Suspended cells were collected from confluent parental cell lines and spun down gently at 900 rpm for 5 min. Pellets were gently dissociated with pipette and resuspended in a serum free media containing 20 ng/ml bFGF, 20 ng/ml epidermal growth factor (EGF), 10 μg/ml insulin, and 4 μg/ml heparin (Leung et al. 2010). Cells were plated on low binding suspension plates with media change every 3–5 days.

### Fluorescence-activated cell sorting (FACS) analysis

CD24^low/−^/CD38^+^ and CD44^+^/CD24^low/−^ expression were used as biomarkers of an enriched population of Lungospheres and mammospheres, respectively (Karimi-Busheri et al. 2012; Rasouli-Nia et al. 2004). Approximately, 0.5 × 10^6^ cells were mixed with FACS phosphate saline buffer (PBS) containing 2 % fetal bovine serum (FBS) and 2 mM ethylenediaminetetraacetic acid (EDTA). Cells were centrifuged at 1,200 rpm and the supernatant was removed and cells were re-suspended in 50 μl FACS buffer containing 2.5 μl CD24-PE and 10 μl CD38 Alexa Flour 488 antibodies. After incubation on ice/dark for 20 min cells were washed and resuspended again in the FACS buffer. Analysis was performed on an Attune Acoustic Focusing Cytometer from Applied Biosystems (Foster City, CA) flow cytometer. As negative control, isotype-matched labeled were used in all experiments.

### Cryopreservation media and procedure

For cryopreservation of the cells we followed modified protocols published earlier by our laboratory (Karimi-Busheri et al. 2010a, b) and protocols published on line (http://www.natureprotocols.com/2006/08/25/neural_stem_cell_culture_neuro.php). Lungospheres were pelleted down by centrifugation at 800 rpm and mixed in TrypLE Express (Invitrogen, Carlsbad, CA) followed by incubation for 10 min. Cells were first mixed with the media (without Me2SO) and then passed through the cell strainer to remove clumps and centrifuged at 800 rpm. Supernatant were removed and cell pellets were gently mixed with cryopreservation media composed of DMEM/F12 media supplemented with non-essential amino acids, penicillin/streptomycin, 2-mercaptoethanol, B27, insulin (5 μg/ml), heparin (4 μg/ml), 15 % Me2SO. Aliquots of 0.5 ml of cell suspensions were transferred into cryovials and kept overnight in a −80 °C freezer and transferred to the liquid nitrogen cryofreezer the next day for storage in vapor phase.

### Reestablishment of frozen lungospheres

After 12 months, vials were removed from the liquid nitrogen and kept at room temperature to reach to ambient temperature. At this stage 10 ml serum free media were added to the tubes followed by 5 min centrifuge at 800 rpm. Supernatants were removed and cells were gently mixed with lungospheres media with sterile pipettes and transferred to low binding plates and incubated at 37 °C/5 % CO_2_. Cells were kept in culture for two passages, approximately 8 day, before being tested in different experiments.

### RNA isolation and microarray analysis

Total RNA was isolated by Absolutely RNA Miniprep kit from Agilent Technologies (La Jolla, CA) from 5 × 10^6^ H460, A549, and their derived lungospheres according to the manufacturer recommendations. Quality of RNA was tested with an OD 260/280 reading of approximately 1.9 and an OD 260/230 great than 1.7 prior to send the samples for microarray analysis at the Core Facility of Sanford-Burnham Institute at La Jolla, CA. Samples were analyzed on an Ilumina Human HT-12 V3 Expression BeadChips and the data were subsequently analyzed on a BeadArray Reader at The Bioinformatics Shared Resources of Sanford-Burnham Medical Research Institute at La Jolla, CA. A *P* value of <0.05 was used as a cutoff to compare the intensity values. The data were finally analyzed based on the basis of *t* tests and fold difference changes in expression level.

### Statistical analysis

Statistical differences between groups were tested by two-tailed Student’s *t* test where a *P* value of < 0.05 or below was considered statistically significant when means of groups of observation were compared. All experiments were performed in triplicate. Statistical and pathway analysis of the microarray data were analyzed through the use of Ingenuity Pathways Analysis (Ingenuity^®^ Systems, www.ingenuity.com). The *P* value was calculated by Fisher’s exact test to determine if there are nonrandom associations among biological function or canonical pathways of fresh versus frozen samples.

## Results

### Expression of lung cancer stem cells markers

**Fig. 1:**
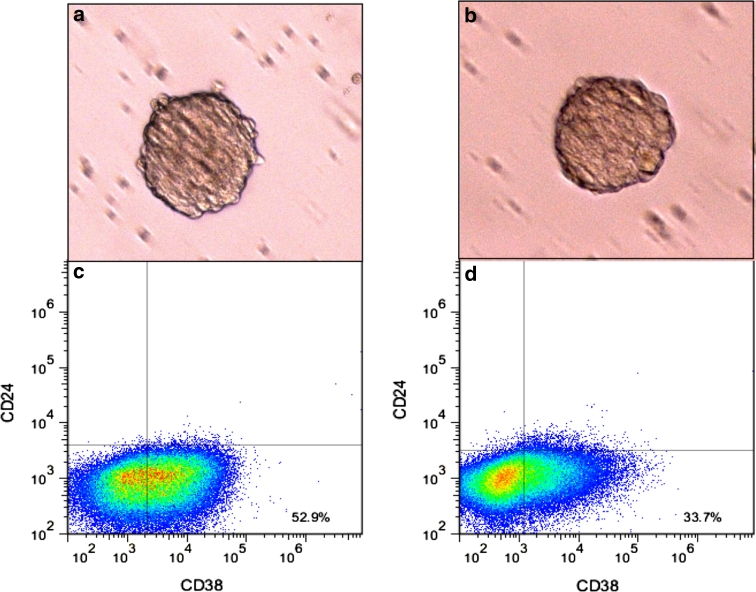
Morphology and biomarkers of H460-derived lung cancer stem cells. Images of fresh (**a**) and two passages post-thaw culture following 12 months cryopreservation of cancer stem cells (**b**). CD24/CD38 expression in fresh (**c**) and post-thaw (**d**) lung cancer stem cells

No morphological differences were observed between fresh and frozen lungospheres as displayed in Figs. 1a, b. We then tested two potential biomarkers of H460-derived lung cancer stem cells, CD24 and CD38. Figure 1c, d represent the expression of CD24/CD38 in fresh and frozen lungospheres, respectively. Frozen lungosphere after thawing and 10 days in culture with two passages were shown to express significantly lower cell surface markers CD 24/CD38 than their freshly grown lungospheres, p = 0.003. Table 1 summarizes the description and function of the genes studied.

**Table 1:** Description of molecular targets

Genes	Description	Function	Refs
CD24	Cell adhesion molecule encodes a sialoglycoprotein that is expressed on mature granulocytes and in many B cells	Modulates B-cell activation responses	Suzuki et al. (2001)
CD38 (cyclic ADP ribose hydrolase)	Marker of cell activation; novel multifunctional ectoenzyme widely expressed in cells and tissues	Cell adhesion, signal transduction and calcium signaling	Malavasi et al. (2008); Zocchi et al. (1999)
ALDH	Catalyze the oxidation of aldehyde	Play a role in the metabolism of many molecules	Alison et al. (2010)
EpCAM (CD326)	Cell surface; carcinoma-associated antigen	Epithelial cell adhesion molecule	van der Gun et al. (2010)
MUC1 (CD227)	Cell surface; encodes a membrane bound, glycosylated phosphoprotein	Cell-cell interactions, signaling, and metastasis	Bafna et al. (2010)
Fas (CD95)	Cell surface; member of the TNF-receptor superfamily	Play a central role in the physiological regulation of programmed cell death. Apoptosis, tumor development, and progression	Gordon and Kleinerman (2009)
HLA-A,B,C	Major histocompatibility complex (MHC) molecules cell-surface receptors	Play a central role in the immune system	Charron (2011)

Summary and function of the genes analyzed for the impact of long-term storage (12 months) on H460- and A549-derived lung cancer stem cells

### Expression of ALDH and EpCAM, potent cancer stem cells markers

**Fig. 2:**
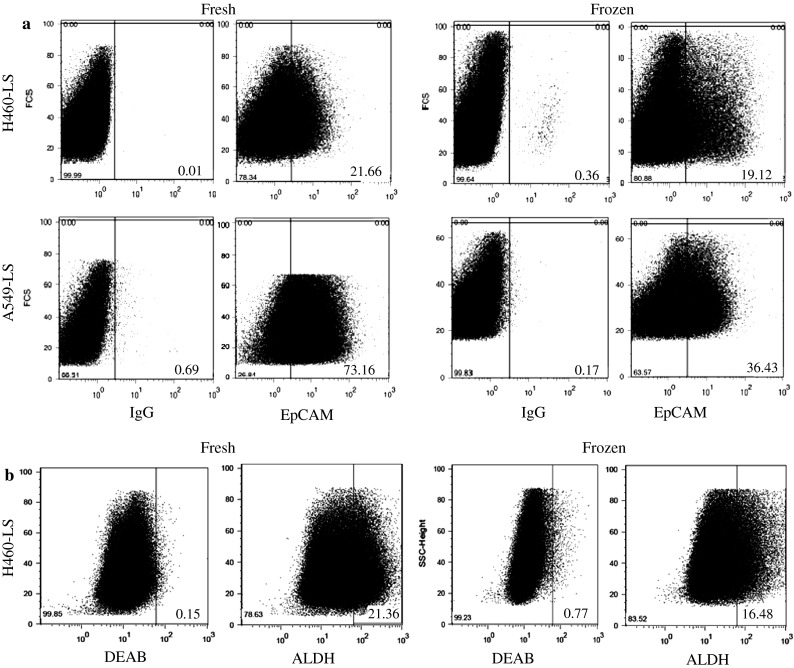

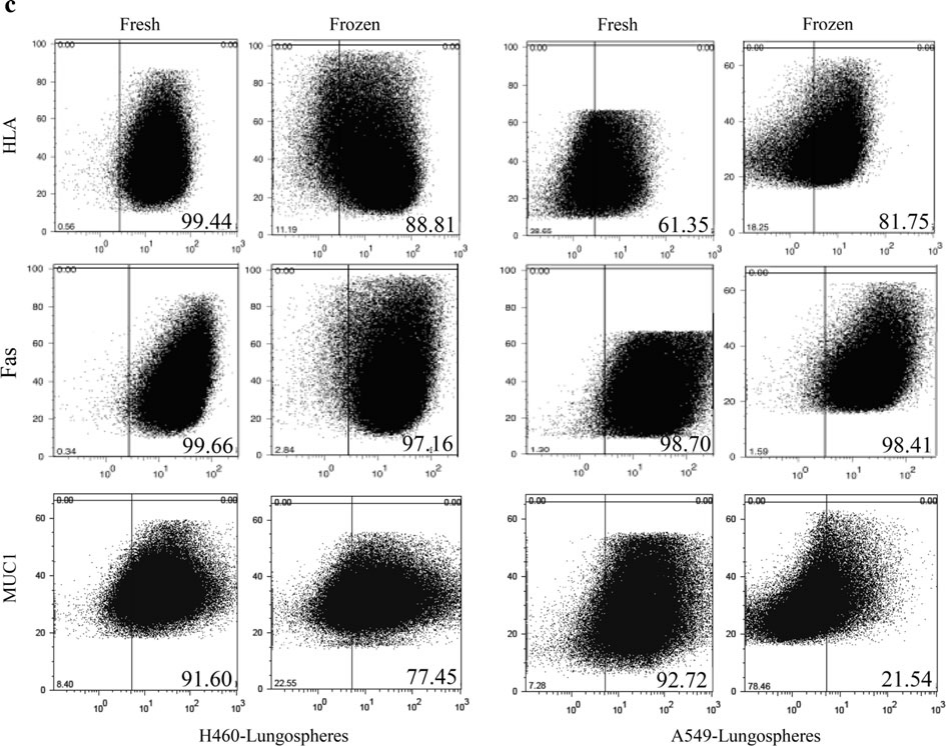
Potent biomarkers of cancer stem cells activity in fresh and frozen cells. **a** The expression of EpCAM in H460 and A549 lungospheres (LS). IgG isotype was used as control. **b** Enzymatic activity of ALDH in H460-derived lungospheres in fresh and 12 months freeze-thaw cells, with DEAB, an inhibitor of ALDH as controls. **c** Analysis of HLA, Fas, and MUC1 by flow cytometry. Expression of three genes in H460- and A549 lungospheres are analyzed in 12 months old cryopreserved cells followed by thawing and two passages in culture and compared with freshly isolated lungospheres of both cell lines

EpCAM expression was analyzed in both H460 lung carcinoma and A549 human lung adenocarcinoma epithelial derived lungospheres. As displayed in Fig. 2a and b, freshly isolated and cultured lungosoheres from both cell lines exhibit a significant higher expression of EpCAM, p = 0.004 and 0.004, respectively (Table 2). The expression of aldehyde dehydrogenase (ALDH), another potent biomarker for cancer stem cells, was only looked in H460 derived lungospheres as A549 adherent cell line express a high level of ALDH that could jeopardize the actual expression of the marker in non-adherent cells (Moreb et al. 2007). Similarly, a statistically significant, p = 0.02, difference exists in the expression of the protein between fresh and freeze-thawed lungospheres as shown in Fig. 2c. Diethylaminobenzaldehyde (DEAB) a specific inhibitor of ALDH was used as control (Muramoto et al. 2010).

**Table 2:** Statistically significant genes showing differential expression

Genes	Cell lines	↑↓	*P* value
CD24/CD38	H460-LS	↓	0.004
EpCAM	H460-LS	↓	0.004
EpCAM	A549-LS	↓	0.02
ALDH	H460-LS	↓	0.02
HLA	H460-LS	↓	0.03
HLA	A549-LS	↑	0.04
Fas	H460-LS	=	0.39
Fas	A549-LS	=	0.44
MUC1	H460-LS	↓	0.05
MUC1	A549-LS	↓	0.0002

The *P* values for seven statistically significant up (↑) and down regulated (↓) genes in post-thaw lung cancer stem cells as compared to fresh cell ± standard deviation of three repeats. No significant changes were observed in Fas gene between fresh and frozen cells as displayed by = in the table. LS represent lungospheres

### Expression of HLA, Fas, and MUC1

We further analyzed the impact of long term preservation on three genes essential for the normal functioning of the cells (Table 1) in H460 and A549 lung cancer stem cells (Fig. 2c). With the exception of Fas gene, the expression of HLA and MUC1 in both cell lines are significantly different between fresh and frozen lungospheres in both cell lines. All cryopreserved cells show a lower expression of the genes than the fresh one with the exception of HLA that the post-thawed cells express a higher level than the fresh one (p = 0.04). A summary of the results of the statistical analysis of all the genes studied are shown in Table 2.

### Microarray analysis

**Fig. 3:**
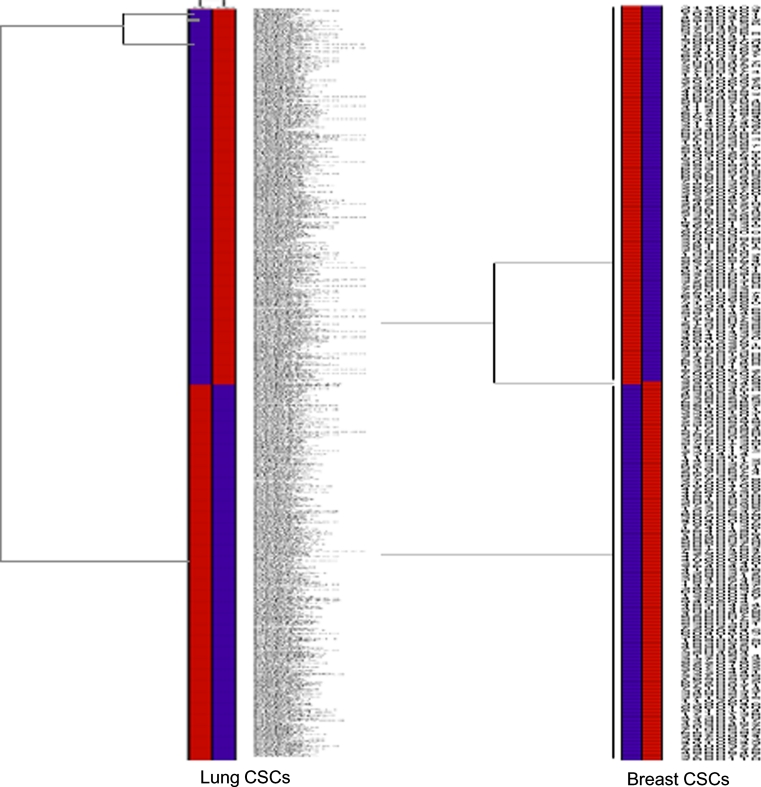
Microarray analysis. RNA microarray analysis of MCF-7 and H460-derived stem cells between fresh and cryopreserved post-thawed cells. The heatmaps representing the data from 4 individual arrays from the twofold significant differences between fresh and cryopreserved post-thawed samples of H460- and MCF7-derived cancer stem cells

To test the impact of long term storage on the global gene expression of lung and breast cancer stem cells we performed a microarray analysis (Fig. 3). In lung cancer stem cells, from 636 genes that underwent a significant minimum twofold change in expression 167 (26.3 %) genes were up-regulated and 469 (73.7 %) genes downregulated. A significantly lower number of genes in breast cancer stem cells were affected under cryopreservation. From a total of 236 genes, 86 (36.4 %) were up-regulated and 150 (63.6 %) down regulated. This indicates that cryopreservation results in approximately 63 % more significant changes in gene expression among lung-derived cancer stem cells than breast MCF7-derived stem cells. The top tenfold change up- and down-regulated molecules are displayed in Table 3.

**Table 3:** Top tenfold change with highest and lowest expression

Mammospheres				Lungospheres			
Molecule	Up	Molecule	Down	Molecule	Up	Molecule	Down
GPX2	8.2	PGM5	10.9	POMC	39.7	TAF15	6.7
AKR1B10	6.4	KLF6	5.8	KRT19	21.3	CHORDC1	4.6
KLK11	4.9	TM4SF1	5.6	GPER	19.8	FAM81A	4.4
S100P	4.4	ANXA1	5.5	CYR61	18.6	PPP3R1	3.9
ID1	4.4	KLF2	5.4	CA9	14.4	SLC1A3	3.8
CA2	3.9	C10ORF81	5.2	REG3G	14.1	CA2	3.8
S100A8	3.9	TNFRSF11B	4.6	NDRG1	13.9	CBX5	3.7
AKR1C3	3.8	DKK1	4.2	NDUFA4L2	13.7	LMNB1	3.6
IFI27	3.7	CYP4B1	4.2	TSC22D3	13.2	TAF5L	3.6
AKR1C4	3.6	RASD1	4.0	MYOM1	12.6	CCDC71	3.5

A twofold change was set to identify molecules up- or down-regulated due to cryopreservation. The numbers under up or down represent the fold change significant differences between fresh and frozen population

### Ingenuity pathways analysis: top biological functions

Through Ingenuity pathway analysis we looked at the statistically significant differences between fresh and prolonged cryopreserved samples of lungospheres and breast cancer stem cells (mammospheres). Various pathways and disorders were analyzed including: diseases and disorders, molecular and cellular function, physiological system development and function, and canonical pathways (Table 4A). The highest number of molecules affected by cryopreservation in both lung and breast cancer stem cells are the molecules involved in genetic diseases; 120 molecules (38.8 %) in mammospheres and 522 molecules (36.9 %) in lungospheres. Within molecular and cellular function, cryopreservation altered gene expression of 76 molecules (25.9 %) in mammospheres and 312 molecules (28.0 %) in lungospheres. When looking at the physiological system development and function we observed 45 molecules (34.9 %) involved in tissue development of mammospheres and 113 (33.2 %) molecules in tissue morphology function of lungospheres with significant up- or down-regulation following long term cryopreservation.

**Table 4:** **A** Distribution of top biological functions among breast and lung cancer stem cells; **B** top canonical pathways affected by freezing in fresh and post-thawed breast and lung cancer stem cells

Category	Breast cancer stem cells		Lung cancer stem cells	
	# Of molecules (%)	*P* value	# Of molecules (%)	*P* value
Diseases & disorders				
Cancer	88 (28.5)	9.06E−13–1.13E−02	381 (26.9)	1.07E−33–6.25E−04
Gastrointestinal disease	32 (10.4)	3.29E−08–2.32E−05	174 (12.3)	8.87E−29–3.70E−04
Reproductive system disease	56 (18.1)	1.54E−07–9.92E−03	217 (15.3)	9.64E−15–4.23E−04
Genetic disorder	120 (38.8)	1.55E−06–1.09E−02	522 (36.9)	2.05E−26–4.55E−04
Organismal injury & abnormalities	13 ( 4.2)	1.45E−05–6.73E−03	–	–
Respiratory disease	–	–	121 ( 8.6)	2.72E−08–2.16E−04
Molecular & cellular function				
Cell death	72 (24.6)	8.89E−10–1.00E−02	312 (28.0)	5.95E−23–5.73E−04
Cell cycle	35 (11.9)	1.38E−05–1.06E−02	174 (15.6)	8.87E−29–3.70E−04
DNA replication, recomb. & repair		–	139 (12.5)	5.00E−14–5.72E−04
Cellular growth & proliferation	76 (25.9)	2.47E−09–1.09E−02	297 (26.7)	2.58E−12–4.64E−04
Cellular development	62 (21.2)	1.39E−07–1.09E−02	–	–
Cellular movement	48 (16.4)	5.02E−09–1.09E−02	192 (17.2)	6.74E−12–5.72E−04
Physiological system development & function				
Tissue development	45 (34.9)	2.85E−06–1.09E−02	108 (31.8)	1.30E−07–5.76E−04
Connective tissue development	–		69 (20.3)	5.51E−06–5.20E−04
Cardiovascular system development	29 (22.5)	3.65E−06–1.09E−02	40 (11.8)	5.72E−06–3.60E−04
Tissue morphology	–		113 (33.2)	9.34E−06–9.40E−04
Embryonic development	27 (20.9)	9.51E−05–1.07E−02	10 (2.9)	1.26E−05–5.27E−04
Endocrine system development	9 (7.0)	1.52E−05–9.99E−03		–
Organismal development	19 (14.7)	3.65E−06–9.37E−03		–

Breast cancer stem cells			Lung cancer stem cells		
Pathway	*P* value	Ratio (%)	Pathway	*P* value	Ratio (%)
B					
Xenobiotics metabolism by cytochrome P450	3.39E−07	11/213 (5.2)	Mitotic roles of polo-like kinase	1.38E−04	12/63 (19.0)
LPS/1L-1 mediated inhibition of RXR function	1.13E−04	10/222 (4.5)	ATM signaling	1.54E−04	11/54 (20.4)
Fatty acid metabolism	8.90E−04	7/196 (0.4)	Cell cycle: G2/M	1.63E−04	10/49 (20.4)
Galactose metabolism	3.60E−03	7/115 (0.4)	Protein ubiquitination	2.56E−04	30/247 (10.9)
Aryl hydrocarbon receptor signaling	4.62E−03	6/155 (0.4)	Antigen presentation	3.74E−04	9/43 (20.9)

The highest ranked canonical pathways significantly affected by prolonged cryopreservation are displayed in Table 4B. None of the pathways are shared among mammospheres and lungosphere. In mammospheres, metabolism of xenobiotics by cytochrome P450 with a *P* value of 3.39E−07 and a ratio of 5.2 % (11 genes out of a 213 total number of molecules that exist in this canonical pathway) represents the most altered canonical pathway. For the lungospheres mitotic roles of polo-like kinase, ataxia telangiectasia mutated (ATM) signaling, and antigen presentation represent the highest number of genes affected in the canonical pathways as displayed in Table 4B.

## Conclusion

We previously discussed the impact of prolonging cryopreservation of breast cancer stem cells on the biological functioning of the cells (Karimi-Busheri et al. 2010a, b). In that report we showed that cryopreservation of breast cancer stem cells does not influence viability, proliferation, basic DNA repair ability, and self-renewal. The only difference we observed was the presence of a significantly higher number of mammospheres undergoing senescence. In this study we focused on the impact of prolonged storage on the molecular integrity and global gene expression patterns of breast and lung cancer stem cells.

Contrary to our previous findings (Karimi-Busheri et al. 2010a, b) that cryopreservation will not generate any observable changes in biological activity of the cells, including proliferation and self-renewal, here we report significant alterations at the molecular level of the post-thawed cells. To prevent the onset of senescence associated with long term culture (Kuilman et al. 2010) and adaptation to culture conditions, post-thawed cells were in culture for two passages before being analyzed. Furthermore, to increase assay reproducibility all the assays and equipments used in our laboratories, in particular our Attune Acoustic Focusing Cytometer from Applied Biosystems (Foster City, CA), are routinely calibrated to the FDA standard of quality to produce consistent results as a requirement for an ongoing lung cancer Phase III clinical trial of belagenpumatucel-L (Lucanix(r)) in our company. Down-regulation of the expression of seven out of nine genes following 12 months freezing could be considered as a dominant pattern in lungospheres following cryopreservation.

As the cell surface proteins play a major role in stem cell fate the majority of genes selected for this study, with the exception of ALDHA1 and HLA, were cell surface markers. As all the cell surface genes studied were down-regulated that might indicate the expected susceptibility of the stem cell membrane to cryopreservation. Previous reports have mainly focused on cell viability but a few reports, including the influence of cryopreservation on amniotic fluid-derived stem cells and human limbal epithelial stem cells (Seo et al. 2011; Vasania et al. 2011) also confirm alterations in molecular profiling of stem cells. Of considerable interest is the result that the cryopreserved human limbal epithelial stem cells express negative immunoregulatory molecules and are non-immunogenic in nature (Vasania et al. 2011). The finding clearly questions the survival of these cells in an allogeneic environment. This could be a significant factor influencing the outcome of any research on stem cells that is intended to be developed for storage, good manufacturing practice, and the manufacturing of clinical grade stem cells.

Global gene expression analysis of fresh versus post-thawed breast and lung cancer stem cells also confirmed a significant amount of down regulation in freeze-thaw cells. Interestingly, gene expression changes in both cell lines are independent from each other and no one gene is shared between the two population sets. There are, however, similarities between both breast and lung cancer stem cell populations in the biological functions distribution and canonical pathways. Genes involved in genetic disorders and cancer rank the top two in the disease category affected by long term storage of the cells. Within the molecular and cellular function category, cell death and cellular growth and proliferation pathways are the most predominantly affected among the two populations. For the physiological system development and function category breast and lung stem cell populations share the highest changes in the tissue development pathway.

Analysis of canonical pathways between breast and lung cancer stem cell reveals interesting results. In post-thawed lung cancer stem cells, the majority of pathways showing significant alteration in gene expression are the pathways involved in cell cycle, mitosis, and ATM that also regulates a number of pathways involved in DNA repair, cell cycle, and apoptosis (Smith et al. 2010). In contrast, in breast cancer stem cells canonical pathways influenced by cryopreservation are mainly associated with metabolism. Our results clearly display the profound impact of long term storage of cancer stem cells under cryopreserved conditions. The influence has a wide spectrum and is cell type dependent. Down-regulation of gene expression is a dominant pattern among post-thawed cells. In adherent and non-stem cell populations greater passaging after thawing could return the cells to normal but in the case of stem cells, with the possibility of senescence (Karimi-Busheri et al. 2010a, b; Kuilman et al. 2010), this could be problematic. Changing freezing conditions and reducing the amount of DMSO (Seo et al. 2011) could be an alternative that remains to be tested in different panels of tissues and cells-derived stem cells.

In conclusion, we observed direct impact of cryopreservation on the molecular integrity of cancer stem cells, in particular on the cell surface membrane. Similar results were also confirmed by global gene expression analysis on two different sets of stem cell populations all indicating a significant down regulation following long term storage of the cells. Our findings could have significant implications in the emerging field of cancer stem cells since cell surface markers, biological functional and canonical pathways play a major role towards understanding cancer initiation and progression.

Finally, we believe current protocols for cryopreservation of cancer stem cells could substantially influence the activity and function of genes and accordingly we encourage employing rigorous research on methodology for freezing and utilizing cancer stem cells following long-term storage. These results are an alarming signals suggesting that cancer stem cells could be more sensitive to long-term storage and recovering process and part of the discrepancies in the results obtained by different investigators on biomarkers and genetic profiling of cancer stem cells might be in fact attributed to the freezing process.
